# Biomedical Properties of a Natural Dietary Plant Metabolite, Zerumbone, in Cancer Therapy and Chemoprevention Trials

**DOI:** 10.1155/2014/920742

**Published:** 2014-06-16

**Authors:** Heshu Sulaiman Rahman, Abdullah Rasedee, Swee Keong Yeap, Hemn Hassan Othman, Max Stanley Chartrand, Farideh Namvar, Ahmad Bustamam Abdul, Chee Wun How

**Affiliations:** ^1^Department of Veterinary Clinical Diagnosis, Faculty of Veterinary Medicine, Universiti Putra Malaysia (UPM), 43400 Serdang, Selangor, Malaysia; ^2^Institute of Bioscience, Universiti Putra Malaysia (UPM), 43400 Serdang, Selangor, Malaysia; ^3^Department of Microbiology, Faculty of Veterinary Medicine, University of Sulaimanyah, Sulaymaniyah, Kurdistan Region, Iraq; ^4^DigiCare Behavioral Research, Casa Grande, AZ, USA; ^5^Institute of Tropical Forestry and Forest Products (INTROP), Universiti Putra Malaysia (UPM), 43400 Serdang, Selangor, Malaysia

## Abstract

Zerumbone (ZER) is a naturally occurring dietary compound, present in many natural foods consumed today. The compound derived from several plant species of the Zingiberaceae family that has been found to possess multiple biomedical properties, such as antiproliferative, antioxidant, anti-inflammatory, and anticancer activities. However, evidence of efficacy is sparse, pointing to the need for a more systematic review for assessing scientific evidence to support therapeutic claims made for ZER and to identify future research needs. This review provides an updated overview of in vitro and in vivo investigations of ZER, its cancer chemopreventive properties, and mechanisms of action. Therapeutic effects of ZER were found to be scientifically plausible and could be explained partially by in vivo and in vitro pharmacological activities. Much of the research outlined in this paper will serve as a foundation to explain ZER anticancer bioactivity, which will open the door for the development of strategies in the treatment of malignancies using ZER.

## 1. Introduction

**Figure 1:**
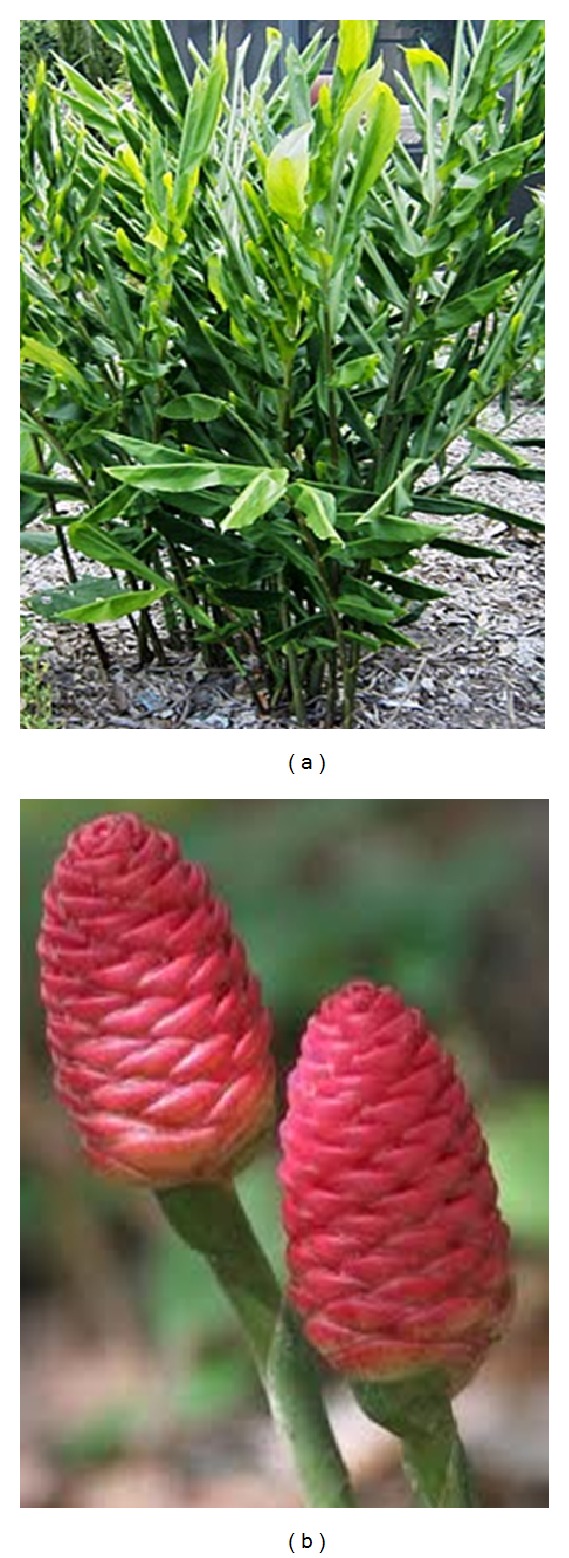
*Zingiber zerumbet* tree (a) and inflorescences (b).

Medical herbs and plant foods such as fruits, vegetables, and spices contain many biologically active phytochemicals that have various health-promoting effects [1]. The Zingiberaceae family found in tropical and subtropical regions of the world and approximately 161 species from 18 genera of this family are found in Peninsular Malaysia [2].* Zingiber zerumbet *(L.) Smith tree (Figure 1(a)), belonging to this family, is an edible ginger, originating in South-East Asia, and has been cultivated for thousands of years as a spice and for medical purposes [3]. Although this plant is known to be indigenous to India and the Malay Peninsula, it is nonetheless distributed in many other countries including Indonesia, China, Bangladesh, Vietnam, Japan, Burma, Nepal, Sri Lanka, Jamaica, and Nigeria and other parts of the globe [4]. This herbal plant is popularly referred to as the pinecone, wild ginger, Asian ginger, or shampoo ginger. It is also called by many other names in different countries, such as* lempoyang* in Malaysia and Indonesia;* parsu kedar, ghatian, and yaiimu* in India [5],* jangliadah* in Bangladesh [6],* hong qui jiang* in China,* haeo dam* in Northern Thailand,* awapuhiin* in Hawaii, and* zurunbah* in the Middle East [3]. Generally, the rhizome and the leaves are used for spice, tea, beverage, and medical purposes, while the milky, mucilaginous substance of the inflorescences (pinecones) (Figure 1(b)) is famously used as a shampoo and natural hair conditioner, especially in Asia and Hawaii [7, 8].

*Zingiber zerumbet *contains several types of phytochemical and is considered as one of the most widely used traditional dietary condiments in various cuisines and beverages throughout Asia, although the essential oil is also used as perfume and in other toiletry articles [9]. Besides its extensive use as a spice, the rhizome particularly has been used in traditional oriental medicine for many human disorders, especially in the treatment of a variety of digestive conditions [10, 11]. The rhizome and oils from the leaves of* Zingiber zerumbet *have been subjected to close chemical scrutiny for their medicinal value [12].

Ginger is generally recognized as safe and is used traditionally in local folk medicine for treatment of nausea, hangovers, asthma, morning and motion sickness, loss of appetite, dyspepsia, diarrhea, colic, cramp, stomach upset, sprain, worm infestation in children, cough and cold, flu, sinusitis, catarrh, congestion, sore throat, migraine headache, toothache, diabetes, bruising, carbuncles, fracture, swelling, rheumatism, arthritis, and chills and fever [13–15].

Presently, rhizome's extract has been extensively studied for its effectiveness in a broad range of biological activities including antimicrobial [16], antipyretic [17], antispasmodic and anticonvulsant [3], antiulcer [18], antioxidant [19], antidiabetic [20], antitumor [21], anticancer [22, 23], anti-inflammatory [24, 25], antinociceptive and analgesic [26, 27], antiallergenic [28], antiangiogenic [29], antidipogenetic [30], antiplatelet aggregation and anticoagulant [31], and hepatoprotective effects [32]. Other studies have shown that consuming the rhizome also exhibits hypolipidemic effect by reducing intestinal cholesterol absorption, which makes it useful for treating heart diseases [33, 34].

**Figure 2:**
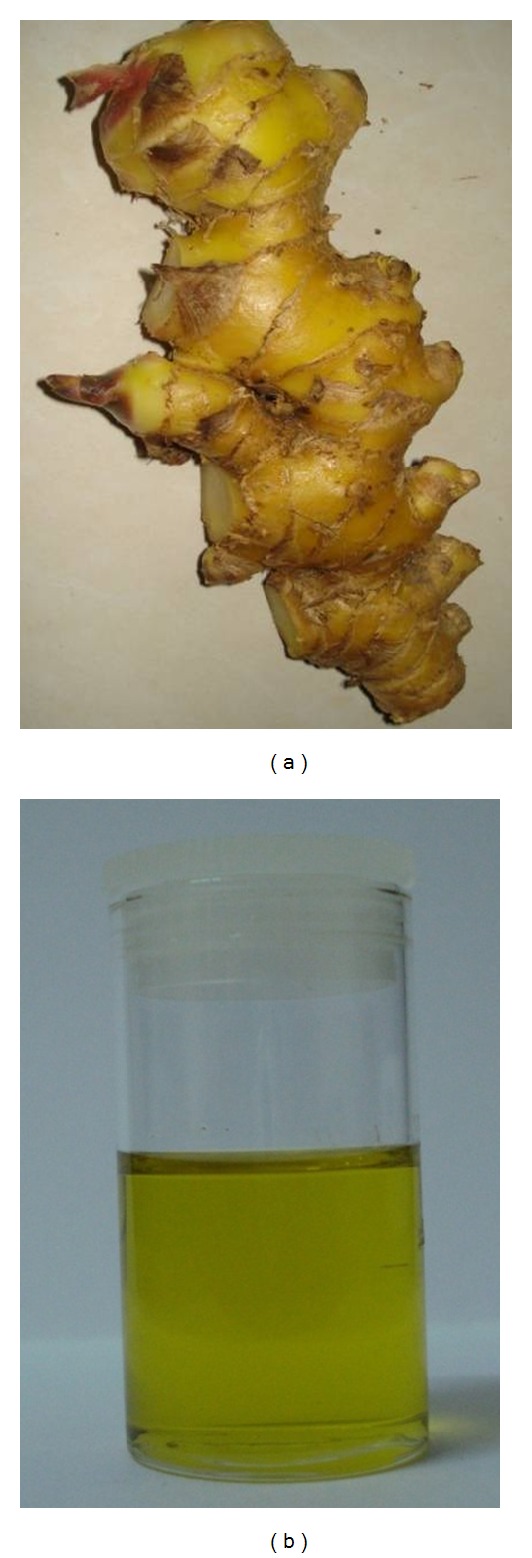
*Zingiber zerumbet *rhizome (a) and essential oil (b).

The essential oil of* Zingiber zerumbet *rhizome (Figure 2(a)) contains approximately 86% sesquiterpenoids [35] while the leaf and rhizome oils (Figure 2(b)) of this plant contain a complex mixture of 29 and 30 compounds, respectively [6]. Many of these compounds are in trace amounts with great variations in their chemical compositions.

**Figure 3:**
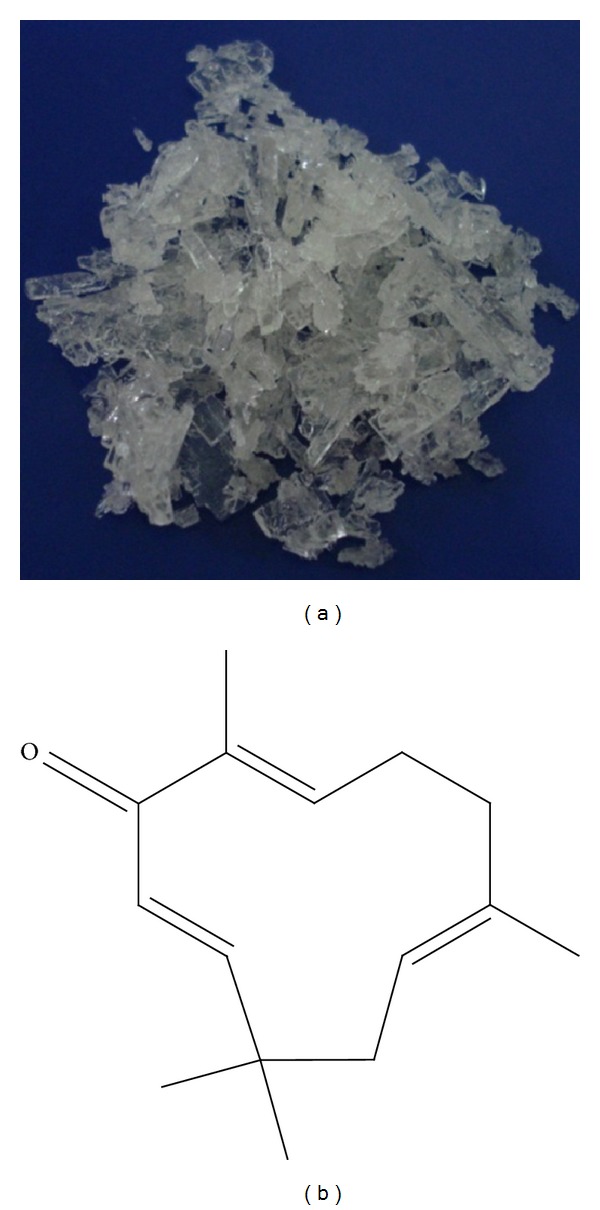
Zerumbone pure crystals (a) and chemical structure (b).

Zerumbone (Figure 3(a)) was first isolated from the essential volatile oil of rhizomes of* Zingiber zerumbet *in 1956 [36], while its chemical structure (Figure 3(b)) was determined in 1960 and later characterized by NMR and X-ray [37]. Zerumbone possesses three double bonds, two conjugated and one isolated, as well as double conjugated carbonyl group in the 11-membrane ring structure [38]. The chemical characteristics of ZER are presented in Table 1 [39–43].

## 2. Plant Sources of Zerumbone

Early investigations in different parts of the world showed that 12.6 to 73.1% of ZER in* Zingiber zerumbet* is in the rhizome oils [44]. The Kerala state in the South Indian accessions reported that in* Zingiber zerumbet* 76.3 to 84.8% of its ZER content is also in the rhizome oils [44]. On the other hand, a silviculture farm in India reported that 1.81% ZER content was found in the rhizome, 0.16% in the root, 0.09% in the leaf, and 0.03% in the flower of* Zingiber zerumbet* [5]. The Penang Malaysian accession recorded the content of ZER in the plant at 68.9% [44]. Another study conducted in the state of Selangor, Malaysia, showed that the ZER content of* Zingiber zerumbet* is 1.3 g/kg rhizome [23]. The oils of* Zingiber zerumbet* from Tahiti Island and Vietnam were also found to be rich in ZER at 65.3 and 72.3, respectively [45, 46]. In Vietnam, ZER was also isolated from the rhizomes of the Vietnamese* Curcuma zedoaria* (Berg.) Roscoe [47]. Other reports on the ginger plant include that by Chane-Ming et al. [48] and Bhuiyan et al. [6] each showing the rhizome to contain approximately 37% of the plant ZER content. The differences in ZER content in the plant are not due to geographic or ecological variations but instead because of differences in ZER chemotype [3].

Other ginger plant species with ZER among their constituents include the* Zingiber amaricans* [49],* Zingiber ottensii* Valeton [50],* Zingiber aromaticum* (17.72%) [51],* Zingiber cassumunar* Roxb. (1%) [52],* Zingiber ottensii* [53], and* Zingiber montanum *[18]. Various other plants also contain ZER; among them are* Curcuma amada* Roxb. [35] from India,* Alpinia galanga *from Sri Lanka [54], and* Xylopia aethiopica* from Ibadan, Southwest Nigeria [55].

## 3. Anticancer Properties of Zerumbone

Several researchers have reported that ZER has both in vitro (Table 2) and in vivo (Table 3) anticancer properties at different concentrations and doses [56]. Zerumbone possesses antiproliferative properties towards several cancer cell lines with minimal effect on normal cells [57–59]. Among the effects of ZER is induction of high intracellular redox potential that can inhibit proliferation of cancer cells [60]. The cytotoxic effect of ZER on the cancer cells appears to be attributed to the versatile *α*,*β*-unsaturated carbonyl group in its structure, which plays an important role in the interaction of the compound with the most biologically active molecules. Clearly the carbonyl group is important for biological activity because *α*-humulene, also found in ginger, lacking in this functional group is virtually and consistently pharmacological inactive [61]. The *α*,*β*-unsaturated carbonyl group in ZER effectively removes the intracellular glutathione (GSH) through the formation of Michael adducts, thus enhancing the potential of intracellular redox (E), resulting in the inhibition of spread of cancerous cells. However, the average intracellular redox potential of normal cells differs from that of cancerous cells; this difference could be the reason for ZER not inducing proliferation of normal cells [60, 61]. Because there is a close link between tumor promotion, inflammation, and oxidative stress, the anti-inflammatory and/or antioxidant compounds could also act as an anticarcinogenic agent [62]. Although the stimulation of neoplastic cell death by ZER was reported to be through the mitochondrial pathway of apoptosis [47], it also exhibits antiproliferative and anti-inflammatory activities through the modulation of NF-*κ*B activity. Zerumbone inhibits NF-*κ*B in association with the sequential suppressions of I*κ*B*α* kinase activity, phosphorylation, and degradation. This compound also inhibits NF-*κ*B-dependent reporter gene expression activated by TNF, TNFR1, TRADD, TRAF2, NIK, and IKK but not by the p65 subunit of NF-*κ*B. Zerumbone also downregulates NF-*κ*B-regulated gene products, including cyclin D1, COX-2, MMP-9, ICAM-1, c-myc, survivin, IAP1, IAP2, XIAP, Bcl-2, Bcl-xL, Bfl-1/A1, TRAF1, and FLIP. These effects lead to the potentiation of apoptosis induced by cytokines and chemotherapeutic agents. The inhibition of these NF-*κ*B-regulated genes expression is in association with the suppression of TNF-induced cancer invasiveness. Thus, it is hypothesized that inhibition of NF-*κ*B and NF-*κ*B-regulated gene expression induced by carcinogens may also represent the molecular basis for cancer prevention and treatment by ZER [63]. Furthermore, it was shown that ZER is a novel inhibitor of CXC chemokine receptor-4 (CXCR4) expression, which mediates homing of tumor cells to specific organs during metastasis, suggesting the potential of the compound in the suppression of metastasis [64]. This receptor has been identified in various tumors including those in the breast, ovary, prostate, gastrointestinal tract, head, neck, bladder, brain, and skin.

### 3.1. Blood Cancer (Leukemia)

It has been shown that ZER effectively suppresses the tumor promoter 12-O-tetradecanoylphorbol-13-acetate- (TPA-) induced superoxide anion (O_2_^−^) generation from NADP oxidase in dimethyl sulfoxide- (DMSO-) differentiated human acute promyelocytic leukemia (HL-60) cells [60]. One study determined the effect of diethyl ether extract of* Zingiber zerumbet *fresh rhizome on cultured P-388D1 cells and in P-388D-bearing CDF mice. This study showed that the extract could induce DNA fragmentation in P-388D1 cells in vitro and significantly prolonged the life of P-388D1-bearing CDF mice. The same result was obtained when the activity of ZER isolated from the same extract was examined in vitro and in vivo [69]. The study further found that ZER inhibited the growth of HL-60 cells, in time- and concentration-dependent manner. HL-60 cell cycle analysis after treatment with ZER showed induction of G2/M arrest and decreased cyclin B1/CDK1 protein level. Using CEM-ss cells as targets, it was shown that ZER increased the number of TUNEL-positive cells and cellular caspase-3 level; the hallmarks of apoptosis [65]. The anticancer effects of ZER seem boundless when it was shown that it inhibits the proliferation of NB4 cell line, derived from acute promyelocytic leukemia cells, through the induction of G2/M phase cell cycle arrest associated with a decline of cyclinB1 protein and phosphorylation of ATM/Chk1. The study indicated that ZER induction of NB4 cell apoptosis was initiated by the expression of Fas (CD95)/Fas ligand (CD95L), concomitant with the activation of caspase-8. At the same time, they found that ZER induced cleavage of Bid, Bax, and Mcl-1 proteins, phosphorylation of Cdc25C and Cdc2 at the Thr48 and Thr14/Tyr15 residues, respectively, degradation of the proteolytic poly-(ADP-ribose) polymerase (PARP), and triggering of cytochrome c release into the cytoplasm [69]. On leukemic cells, ZER is cytotoxic to human myeloid (KBM-5) [67], mouse myelomonocytic (WEHI-3B) [126], and human acute lymphoblastic leukemic (Jurkat) cell lines [66]. Zerumbone also regulates expression of apoptotic biomarkers in BALB/c mice model of acute myelocytic leukemia via the mitochondrial intrinsic pathway [70].

### 3.2. Skin Cancer

Zerumbone suppressed 7,12-dimethylbenz[*α*]anthracene- (DMBA-) and TPA-induced initiation and promotion of skin tumors in female ICR mice. Using RT-PCR, it was shown that ZER enhances expression of manganese superoxide dismutase (MnSOD), glutathione peroxidase-1 (GPx-1), glutathione S-transferase-P1, and NAD (P) H quinine oxidoreductase (NQO1) mRNA in the epidermis while diminishing TPA-induced COX-2 protein expression and phosphorylation of extracellular signal-regulated kinase 1 and 2 (ERK1/2) [127]. The phorbol ester-induced papilloma formation in mouse skin can also be inhibited by ZER [110]. Recently, it was found that ZER induces heme oxygenase-1 expression in female HR-1 hairless mouse skin and cultured murine epidermal (JB6 Cl4) cells, through the activation of Nrf2 [78]. More recently, ZER was found to induce apoptosis and autophagy in human (WM1552C) and murine (B16-F0) melanoma cell lines [128]. Zerumbone also significantly reduced tumor mass and lung metastasis in B16-F0 bearing C57 BL/6 male mice through the activation of Akt and MAPK and suppression of NF-*κ*B activation [77].

### 3.3. Liver Cancer

Zerumbone was also found to inhibit the proliferation of nonmalignant Chang liver cell line [129], while being innocuous to the normal human liver (WRL-68) cells [86]. DNA fragmentation and apoptosis induced by ZER is by way of up- and downregulation of Bax/Bcl-2 proteins independent of functional p53 activity in the liver adenocarcinoma (HepG2) cell lines. In vivo, ZER inhibits diethyl nitrosamine (DEN) and dietary 2-acetylaminofluorene- (AAF-) induced Sprague Dawley rat hepatocarcinogenesis. This effect was suggested to be through the reduction of oxidative stress, inhibition of cancer cell proliferation, and induction of mitochondria-regulated apoptosis of liver cancers [105].

### 3.4. Cervical Cancer

Zerumbone is known to exhibit an antiproliferative effect on human cervical cancer (HeLa) cell line [87]. In diethylstilboestrol- (DES-) induced mice cervical interepithelial neoplasia (CIN), ZER caused overexpression of proapoptotic protein, Bax [88, 130].

When ZER and cisplatin were used in combination, the cervical cancer in BALB/c mice was suppressed through the modulation of serum interleukin-6 [131]. One experiment was conducted on pregnant BALB/c rats treated with DES to develop cervical intraepithelial neoplasia. When the progenies were treated with different doses of ZER, histological examination revealed that ZER had inhibited the cervical dysplasia from developing into more severe dysplasia [89].

### 3.5. Colon Cancer

Zerumbone was shown to inhibit the proliferation of human colonic adenocarcinoma (LS174T, LS180, COLO205, and COLO320DM) cell lines in a dose-dependent manner, while the growth of normal human colon (CCD-18Co) fibroblasts and normal human dermal (2F0-C25) cells was less affected [90, 110]. The effect of ZER on human colorectal cancer (HCT116) cells was via potentiation of TRAIL-induced apoptosis [90, 91] as indicated by the expression of TRAIL death receptor (DR) 4 and 5. The subsequent effects were activations of caspase-8, caspase-9, and caspase-3 and PARP and downregulation antiapoptotic protein c-FLIP expression and activation of ERK in a time-dependent manner. The RT-PCR assay showed that ZER markedly induced the expressions of IL-1*α*, IL-1*β*, IL-6, and TNF-*α* in human colon adenocarcinoma (Caco-2, Colo320DM, and HT-29) cell lines, in concentration- and time-dependent manners [110]. Developing azoxymethane- (AOM-) induced rat colonic aberrant crypt foci (ACF) in male F344 rat can be significantly inhibited by ZER treatment through suppression of COX-2 expression, cell spreading activity of colonic mucosa, and induction of phase II detoxification enzymes [104]. Similarly, using ACF as a preneoplastic marker, ZER was shown to suppress AOM-induced colon cancer in male Sprague Dawley rats [101]. Zerumbone inhibited the multiplicity of colonic adenocarcinoma induced by AOM, potentiated apoptosis, and suppressed NF-*κ*B and HO-1 expressions in male ICR mice [102].

### 3.6. Bile Duct Cancer

**Figure 4:**
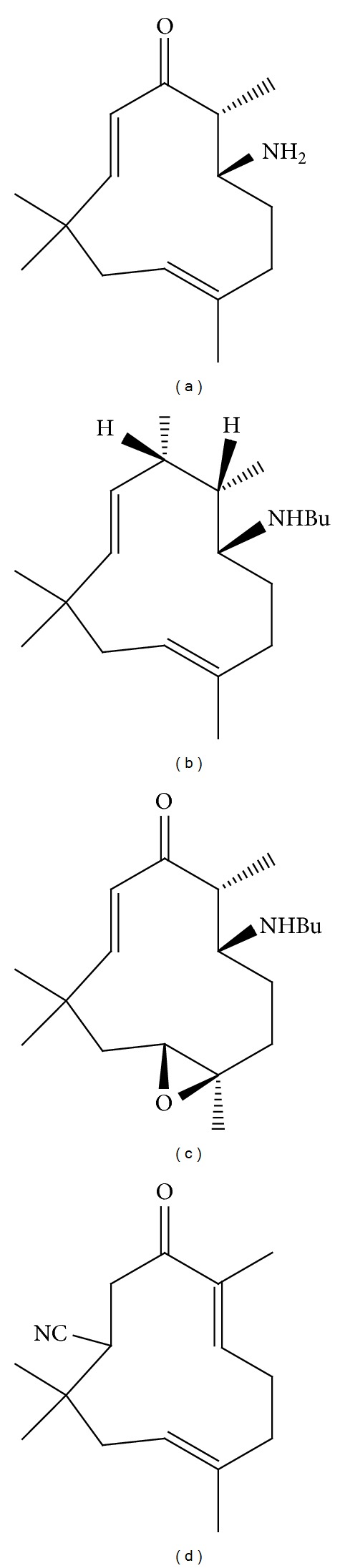
Zerumbone derivatives. (a) (±)-*[*6*E*, 10*E]-3amino-2,6,9,9-tetramethylcloundeca-6,10-dienone *(5), (b) (±)-*[*6*E*, 10*E]-3-butylamino-2,6,9,9-tetramethylcloundeca-6,10-dienol *(10),(c) (±)-*[*10*E]-3-butylamino-6,7-epoxy-2,6,9,9-tetramethylcloundeca-10-enone *(14), and (d) (±)-*[*2*E*, 6*E]-10-cyano-2,6,9,9-tetramethylcloundeca-2,6-dienone *(20).

Amine 5 derived from ZER showed potent antiproliferative activity against cholangiocarcinoma (CCA) cell line and poorly differentiated adenocarcinoma (KKU-100). However, amine 5 and other ZER derivatives (10, 14, and 20) (Figure 4) showed lesser cytotoxicity toward other CCA cell lines including squamous (KKU-M139) cell carcinoma, moderately differentiated adenocarcinoma (KKU-M156), adenosquamous carcinoma (KKUM213), and moderately differentiated adenocarcinoma (KKU-M214) [92].

### 3.7. Breast Cancer

**Figure 5:**
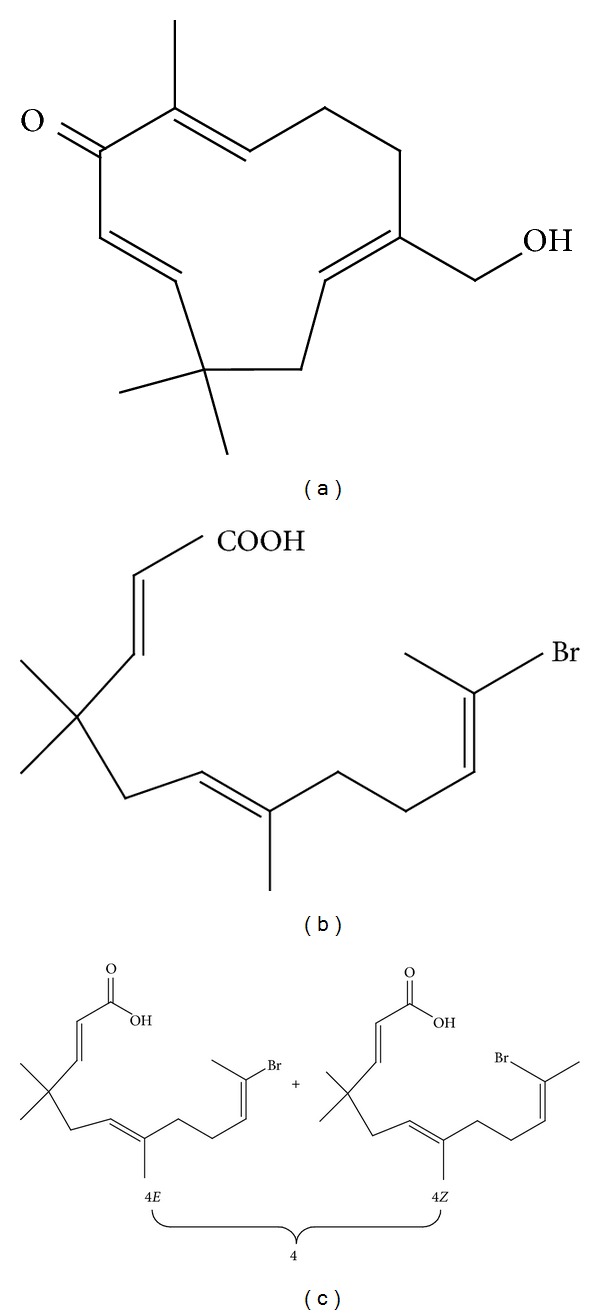
Zerumbone imidazole and ring opening derivatives. (a) Parent alcohol 8* (2E,6Z,10E)-13-Hydroxy-2,9,9-trimethylcycloundeca-2,6,10-trienone,* (b) NH0891 ([*2E,6E*,*10E/Z0*]-11-bromo-4,4,7-trimethyl-2,6,10-dodecatrienoic acid), and (c) 4 (10*E*/10*Z* = 3/2).

In breast cancers, ZER caused G2/M phase cell cycle arrest associated with downregulation of cyclin B1, Ddk1, Cdc25C, and Cdc25B and Bax/Bak-mediated apoptosis in human breast cancer (MDA-MB-231 and MCF-7) cells and retarded growth of MDA-MB-231 xenografts in vivo [76]. In addition, its derivative, parent alcohol 8 (2*E*,6*Z*,10*E*)-13-Hydroxy-2,9,9-trimethylcycloundeca-2,6,10-trienone (Figure 5(a)) significantly displayed antiproliferative effect towards human breast cancer (MCF-7) cell line [97]. The inhibition of mammary tumor growth in LA7-bearing Sprague Dawley rats was via Wnt/*β*-catenin signaling pathway [109].

### 3.8. Ovarian Cancer

The antiproliferative effect of ZER towards human ovarian cancer (Caov-3) cell line is dose dependent and time dependent. Zerumbone also effectively suppressed tumor promoter TPA-induced superoxide anion (O_2_^−^) generation from xanthine oxidase (XO) in Chinese hamster ovary (AS52) cells (CHO) [132], while even at high concentrations it does not adversely affect normal cultured CHO [93].

### 3.9. Pancreatic Cancer

Zerumbone is a novel inhibitor of Jak2/Stat3, which inhibits promigratory gene expression, growth, and migration of human pancreatic carcinoma (PaCa) [94]. It also inhibits CXCL12-induced spread of pancreatic (PANC-28, MIA PaCa-2, and AsPC-1) tumors [64]. The antipancreatic cancer effect of ZER is facilitated by the inhibition of cancer angiogenesis through the inhibition of NF-*κ*B and NF-*κ*B-dependent proangiogenic gene products [96]. The inhibition and apoptosis of human pancreatic carcinoma cell lines (PANC-1 and SW1990) were via p53 signaling pathway [95].

### 3.10. Lung Cancer

The nonsmall lung adenocarcinoma (H1299) cell can be suppressed by ZER, while its derivative, the parent alcohol 8 (*2E*,*6Z*,*10E*)-13-Hydroxy-2,9,9-trimethylcycloundeca-2,6,10-trienone, is one of the most potent cytotoxic compounds against human small cell lung carcinoma (NCI-H187) [97]. Zerumbone also effectively inhibited proliferation, multiplicity of lung adenomas induced by NNK, potentiated apoptosis, and suppressed NF-*κ*B and HO-1 expressions in female A/J mice [133].

### 3.11. Renal Cancer

Human embryonic kidney carcinoma (A293) cell [64] and kidney epithelial (MDBK) cell line [129] proliferation was found to be inhibited by ZER treatment. Zerumbone could also protect irradiation-induced cell apoptosis and DNA damage, partly through the activation of the Keap1/Nrf2/ARE pathway in human kidney embryonic (HEK 293) cells [98]. The ZER derivative, parent alcohol 8 (*2E*,*6Z*,*10E*)-13-Hydroxy-2,9,9-trimethylcycloundeca-2,6,10-trienone, showed nonsignificant cytotoxicity toward normal monkey kidney (Vero) cell line [97].

### 3.12. Brain Cancer

Zerumbone can induce human glioblastoma multiforme (GBM8401) cell apoptosis via inhibition of the IKK*α*-Akt FOXO1 cascade [99].

### 3.13. Prostate Cancer

Zerumbone induced cytotoxicity and significant PARP cleavage in human prostate cancer (DU145) cell line through the inhibition of Jak2/STAT3-mediated signaling pathways [134].

### 3.14. Gastric Cancer

Zerumbone inhibits tumor angiogenesis in human gastric adenocarcinoma (AGS) cells of via reduction of VEGF production and NF-*κ*B activity [135].

### 3.15. Oral Cancer

Parent alcohol 8 (*2E*,*6Z*,*10E*)-13-Hydroxy-2,9,9-trimethylcycloundeca-2,6,10-trienone is one of the most powerful compounds inducing cytotoxicity of human oral cancer (KB) cells [97].

### 3.16. Head and Neck Cancer

Expression of CXCR4 and invasion and metastasis of human tongue squamous (SCC4) cell carcinoma can occur with ZER treatment [64]. Similarly, ZER inhibited the NF-*κ*B- and NF-*κ*B-regulated gene expression induced by various carcinogens and inflammatory stimuli, such as TNF, okadaic acid, cigarette smoke condensate, phorbol myristate acetate, and H_2_O_2_. It also suppressed I*κ*B*α* kinase activity, phosphorylation, and degradation and p65 phosphorylation, nuclear translocation, and acylation in human squamous (LICR-LONHN5) cell carcinoma line [63].

### 3.17. Pharyngeal Cancer

Zerumbone inhibited NF-*κ*B and I*κ*B*α* kinase, suppressed antiapoptotic and metastatic gene expression, upregulated apoptosis, and inhibits proliferation of human hypopharyngeal carcinoma (FaDu) cells [63].

## 4. Anti-Inflammatory Activity

Zerumbone has been shown to possess anti-inflammatory properties [25, 26]. Oral ZER treatment suppressed dextran sodium sulfate- (DSS-) induced acute ulcerative colitis (AUC) in female ICR mice. The anti-inflammatory effect of ZER was reflected by the significant lowering of the inflammatory biomarkers, IL-1*β*, TNF-*α*, and PGE2 [103]. In a female ICR mouse ultraviolet B (UVB) photokeratitis and cataractogenesis model, dietary ZER prevented corneal damage by inhibiting NF-*κ*B, iNOS, and TNF-*α* expression with concomitant reduction of malondialdehyde (MDA) and increase of glutathione (GSH) and GSH reductase (GR) levels [111, 112]. Moreover, ZER inhibited iNOS and COX-2 expression and release of TNF-*α* in a mouse macrophage (RAW264.7) cell line treated with lipopolysaccharide (LPS) and IFN-*γ*. Zerumbone also inhibited the NO/O_2_^−^ generation in inflammatory leukocytes [61, 103]. Oral feeding of ZER compound reduced the inflammatory process in collagen-induced osteoarthritis (OA) in Sprague Dawley rats. The treatment caused a significant reduction in the number of major histocompatibility complex (MHC) type II cells expressions in the affected synovial membrane and thus reducing accumulation of antigen presenting type A cells in arthritis [115]. In a rat knee osteoarthritis model, induced with monosodium iodoacetate (MIA), oral administration of ZER improved the densities of protein gene products (PGP), calcitonin gene-related peptide (CGRP), and neuropeptides-Y (NPY) immunoreactive nerve [116, 117].

In male Wistar rats, ZER suppressed cholecystokinin octapeptide- (CCK-8-) induced acute pancreatitis with significant reduction in serum amylase and lipase, cytosolic IL-6, iNOS, Mn- and Cu/Zn-SOD activities, and TNF-*α* concentration [113]. In these rats ZER treatment attenuates the severity of acute necrotizing pancreatitis and pancreatitis-induced hepatic injury via the inhibition of NF-*κ*B and downregulation of ICAM-1 and IL-1*β* expressions [114].

## 5. Antioxidant Activity

The antioxidant activity of ZER has been reported to occur through the attenuation of reactive oxygen (RO) and generation of nitrogen species [136]. Thus, it is plausible that the potential of ZER as an agent against cancer-related inflammation may be mediated through its antioxidant activity. The ability of ZER to stimulate phase II detoxification enzymes was determined in the RL34 cells, a normal rat liver epithelial cell line. Induction of phase II enzymes is known to protect cells and tissues against toxicity and chemical carcinogenesis, particularly in the early phase. The effect of ZER on the stimulation of glutathione S-transferase is dose- and time-dependent and causes considerable increase in the level of the GSTP1-1 protein. Zerumbone also elicited significant induction in the nuclear localization of Nrf2, a transcription factor that binds to the antioxidant response element (ARE) of phase II enzyme genes, activating expression of phase II enzyme genes. Among the phase II enzyme involved in the activation are *γ*-glutamylcysteine synthetase (GCS), glutathione peroxidase (GPx), and HO-1. These enzyme systems, through their conjugation reactions, play important roles in the metabolic inactivation of pharmacologically active substances, thus minimizing cell damage [85].

## 6. Immunomodulatory Activity 

Zerumbone has effect on the proliferation, cell cycle progression, and induction of cytokine (IL-2 and IL-12) of immune cells in vitro. This was shown by the proliferation of ICF mice thymocytes and splenocytes and human peripheral blood mononuclear cells (PBMC). Using flow cytometry, ZER treatment was shown to cause the highest population of PBMC to enter G2/M phase [73]. This study showed prominent upregulation of IL-2 and IL-12 in activated lymphocytes after ZER treatment.

## 7. Other Biomedical Properties of Zerumbone

### 7.1. Hepatoprotective Activity

Zerumbone was shown to have hepatoprotective properties in ethanol-induced liver injury in male Sprague Dawley rats, while ZER pretreatment extensively reduced fatty liver development in these rats [106]. Similar ZER has healing effects in paracetamol-induced hepatotoxicity in male Sprague Dawley rats as indicated by the corresponding reductions of alanine aminotransferase (ALT), aspartate aminotransferase (AST), and alkaline phosphatase (ALP) blood concentrations in the treated rats [87].

### 7.2. Antiatherosclerotic Activity

Zerumbone is a phytochemical with potential for the regulation of atherosclerosis because it suppresses TPA-induced oxidized low density lipoprotein (LDL) receptor-1 (LOX-1) mRNA expression in THP-1 human monocyte-like cells and in differentiated colonic adenocarcinoma (Caco-2) cells. A key event in the development of atherosclerosis is the unregulated uptake of oxidized LDL via scavenger receptors (SR), which are integral membrane proteins. Zerumbone reduces the expression of several subclasses of the macrophage SR such as SR-A, SR-PSOX, and CD36, leading to the inhibition of uptake of DiI-acLDL, a modified LDL. Downregulation in the expression of SR by ZER was postulated to be partly attributed to the inhibition of transcriptional activities of activator protein-1 and NF-*κ*B [75]. In rabbits fed cholesterol-rich diet, oral ZER treatment significantly decreased or averted early atheroma plague formation and development via reduction in monocytes and/or macrophages migration, aggregation, and smooth muscle cells proliferation. In a rabbit atherosclerosis model, ZER was also shown to repair endothelial dysfunction [124].

### 7.3. Antinociceptive Activity

Significant antinociceptive effects of intraperitoneal ZER were observed in adult male BALB/c mice. The results of this study indicated that ZER possesses considerable marginal and central antinociceptive effects at various dosages [27]. The production of antinociception in the mice model suggests significant involvement of L-arginine-nitric oxide-cGMP-PKC-K+ ATP channel pathways, the TRPV1 and kinin B2 receptors [118].

### 7.4. Antimicrobial Activity

**Figure 6:**
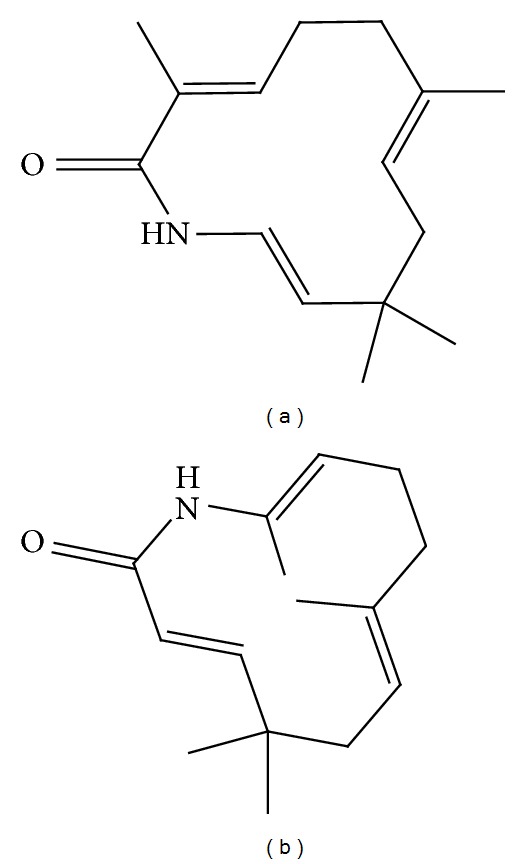
Zerumbone derivatives. (a) Azazerumbone 1, and (b) azazerumbone 2.

Zerumbone and its derivatives such as 410*E*/10*Z* = 3/2 and NH0891 (Figures 5(b) and 5(c)) were found to be selective inhibitors of gram-positive bacteria,* Bacillus subtilis* 168 growth. It was suggested that the new haloolefinic acids synthesized by the cleavage of the C1-C2 bone of ZER inhibits growth of gram-positive bacteria by inhibiting YycG histidine kinase [137, 138]. Zerumbone also inhibits* Salmonella choleraesuis, *a gram-positive bacteria while not affecting the viability of* Escherichia coli* [139]. Similarly, ZER and its synthetic analogues (azazerumbone 1 and azazerumbone 2) (Figure 6) exhibited strong protection against sodium azide-induced mutagenicity of* Salmonella typhimurium* (TA 98 and TA 1531) strains. Among the bacteria tested,* Bacillus cereus* was most sensitive to these analogues [140].

Other antipathogen effects of ZER include inhibition of human immunodeficiency virus (HIV) activity [33] and antifungal activity towards* Rhizoctonia solani,* the damping-off pathogen [52].

Zerumbone was reported to have antimalarial activities by inhibiting propagation of* Plasmodium falciparum *[141]. Exposure of the nematode* Caenorhabditis elegans* to ZER increased expression of HSP16.41 mRNA, suggesting that ZER can increase the survival of nematodes after heat-shock treatment.

In lipid metabolism, ZER improved dyslipidemia by modulating expression of genes involved in the lipolytic and lipogenic pathways of a diet-induced hyperlipidemic animal model [125]. This study suggests that ZER is beneficial to patients with hypercholesterolemia and hypertriglyceridemia. Another study showed that ZER attenuated nonalcoholic fatty liver disease, improved insulin sensitivity, decreased lipogenesis, and increased lipid oxidation in male golden Syrian hamster [108]. Zerumbone also seems to be beneficial in alleviating symptoms of renal dysfunction. Treatment of female Sprague Dawley rats with cisplatin-induced renal disease with ZER had reduced toxicity and organ damage via the preservation of antioxidant glutathione and prevention of lipid peroxidation [123].

Zerumbone induces genotoxic and cytotoxic effects on cultured human peripheral blood lymphocytes [71], CHO cells, and rat bone marrow polychromatic erythrocytes (PCEs) [74, 142]. In fact highly concentrated ZER could cause substantial increase in the frequency of micronuclei in these cells. This study suggests that there are safety issues in the development of ZER as a potential therapeutic compound, because very high doses of ZER may produce adverse effects.

Finally, there is evidence that ZER may be useful in the treatment of Alzheimer's disease. This was suggested by a recent study that showed ZER inhibits acetylcholinesterase [143]. The enzymolytic effect of ZER towards AChE (acetylcholinesterase) could be the basis for the development of ZER in the treatment of Alzheimer's disease.

## 8. Discussion

Many natural compounds possess various and significant biological activities. Thus traditionally these compounds are included in the diet of many Asian societies because they are not only nontoxic but also beneficial to health [144]. However, there is a dearth of scientific and clinical evidence supporting effectiveness, usefulness, and safety of herbal compound used in traditional medicine. Because of lacking evaluation of the toxicity and negative reactions of medicinal herbs, the use of natural compounds may prove unsafe.

Malaysia, with its tropical rainforests, is blessed with high biodiversity. The Malaysian forest is an enormous potential source of chemicals and metabolites that can be developed into new agents or novel drugs for treatment of chronic diseases [145]. The jungles of South East Asia have provided more than 6,500 different plants that have been used in the treatment of various illnesses particularly cancers [146]. The South East Asians seemed to have lower risks for development of cancers including colon, gastrointestinal, prostate, and breast cancers compared to Westerners [147]. It is probably the practice of regular consumption of natural plant products that contributes to the lower incidence of these debilitating diseases in the South East Asians.

Recently, in our laboratory, ZER was made soluble by incorporating in the cyclodextrin complex. The production of the ZER-cyclodextrin complex enabled ZER to be formulated as an encapsulated natural compound ready for use, either as an injectable solution or delivered orally as an anticancer product [148, 149]. The usefulness of encapsulated ZER complex as potential anticancer is worth future exploration through preclinical and human clinical trials to determine efficacy and safety of the product for human use. More recently we also encapsulated ZER into a nanostructured lipid carrier (NLC) using the high pressure homogenization (HPH) technique. The physiochemical properties, entrapment efficiency, storage stability, in vitro release, and cytotoxic effect of this formulation against human acute lymphocytic leukemia (Jurkat) cell line were studied and showed promising results. Our study also showed that ZER-loaded NLC can be further developed as a drug delivery system for cancer therapy [23, 66]. This new approach to using a natural metabolite in innovative delivery systems would seemingly be an alternative and new approach in the treatment of cancers [72].

This review has clearly indicated that ZER from* Zingiber zerumbet *Smith possesses various beneficial in vitro and in vivo biological activities. The findings from all the researches reviewed in this paper are conclusive evidences that ZER is a strong potential candidate for anticancer compound. There is need to conduct animal studies and human clinical trials to ascertain the efficacy, usefulness, and safety of this compound as an intended pharmaceutical drug.

## Figures and Tables

**Table 1:** Characteristic features of zerumbone.

Characters	Description
Natural occurrence	*Zingiber *species
Chemical class	Sesquiterpene
Chemical formula	(2E, 6E, 10E)-2,6,9,9-tetramethylcycloundeca-2,6,10-trien-1-one
Molecular formula	C_15_H_22_O
Chemical structure	Three-double bond (two conjugated and one isolated), *α*,*β*-unsaturated carbonyl group, and a double conjugated carbonyl group in 11-membered ring structure
Molecular weight	218.3 dalton
Flashing point	272°F
Boiling point	321-322°C at 760 mmHg
Melting point	65.3°C
Vapor pressure	0.000295 mm/Hg at 25°C
Purity	92–100%
Appearance	Solid white crystals or powder
Short term storage	+4°C
Stability	Stable for at least 2 years when stored at −20°C
Solubility	Completely soluble in ethanol, DMSO, while solubility in water is approximately 1.296 mg/L at 25°C
Extraction	Mainly isolated from fresh rhizomes by hydrodistillation (steam distillation) and recrystallization methods
Usage	For researches and medical purposes, not for flavor or fragrance

**Table 2:** In Vitro biological effects of zerumbone.

Organ	Cell line	Biological effect of ZER
Blood	Human acute lymphocytic leukemia (CEM-ss) [65]	Induces apoptosis and DNA internucleosomal degradation activate caspase-3
	Human acute lymphoblastic leukemia (Jurkat) [66]	Induces G2/M cell cycle arrest Induces intrinsic apoptotic pathway via activation of caspase-3 and caspase-9, cytochrome c release from mitochondria, and PARP cleavage
	Human chronic myeloid leukemia (KBM-5) [63]	Induces cytotoxicity
	Human acute promyelocytic leukemia (HL-60) [61, 67]	Suppresses TPA-induced superoxide anion generation from NADPH oxidase Induces G2/M cell cycle arrest in time- and concentration-dependent manner Decreases cyclin B1/CDK1 protein level
	Human acute promyelocytic leukemia (NB4) [67]	Induces G2/M cell cycle arrest associated with decline of cyclin B1 protein and phosphorylation of ATM/Chk1, induced apoptosis via expression of Fas (CD95)/Fas ligand (CD95L), with the activation of caspase-8
	Human acute myelocytic leukemia (U937) [67]	Antagonizes action of DDT and TCDD by upregulating the expressions of COX-2 and VEGF mRNA
	Human acute lymphoblastic leukemia (MOLT4), human acute lymphocytic leukemia (OKM-2T), and human chronic myelocytic leukemia (K562 and KT-1) [67]	No cytotoxicity at concentration of 10 *μ*M
	Human peripheral blood multiple myeloma (U266) [68]	Suppresses CXCR4 expression
	Murine lymphoid neoplastic (P-388D1) [69]	Causes DNA fragmentation and growth inhibition
	Murine acute myelocytic leukemia (WEHI-3B) [70]	Induces G2/M cell cycle arrest and apoptosis
	Normal human umbilical vein endothelial cell (HUVEC) [67]	Does not inhibit proliferation at concentration of 10 *μ*M
	Normal human primary mononuclear cells (PBMCs) [71, 72]	No cytotoxicity (1–100 µg/mL) Cytotoxic at high doses (40–80 *μ*M)
	Mice thymocytes and splenocytes human PBMC [73]	Stimulates time- and dose-dependent proliferation of mice cells and human PBMC Upregulates human cytokine (interleukin, IL-2 and IL-12) immunomodulatory
	Human peripheral blood lymphocytes (PBL) Al [74]	Cytotoxic but not clastogenic at 40 and 80 *μ*M, Does not induce chromosomal aberration and micronuclei formation
	Lymphoblastoid (Raji) cells	Suppresses tumor promoter 12-*O*-tetradecanoylphorbol 13-acetate- (TPA-) induced activation of Epstein-Barr virus
	Human monocyte-like cells (THP-1) [75]	Suppresses TPA-induced LOX-1 mRNA expression Attenuates expression of SR-A, SR-PSOX, and CD-36 and led to block DiI-AcLDL uptake Inhibits AP-1 and NF-*κ*B transcriptional activity
	Normal murine macrophages 9RAW264.7) [68]	Markedly diminishes inducible nitric oxide synthase (iNOS) and cyclooxygenase (COX)-2 expression Suppresses free radical generation and inhibits tumor necrosis factor (TNF)-*α* release Induces phase II drug metabolizing enzymes GSTP1 and NQO1 mRNA expressions
	Immortalized mouse embryonic fibroblasts (SV40) [76]	Not cytotoxic
	Human whole blood [31]	Inhibits platelet aggregation induced by arachidonic acid (AA), collagen, and ADP
Skin	Human melanoma (WM1552C) [77]	Induces apoptosis and autophagy
	Murine melanoma (B16-F0) [77]	Induces apoptosis and autophagy
	Normal human dermal fibroblast (2F0-C25) [77]	Not cytotoxic at a concentration of 13 *μ*M
	Murine epidermal cells (JB6 Cl41) [78]	Induces heme oxygenase-1 expression through activation of Nrf2
Liver	Human liver adenocarcinoma (HepG2) [79]	Induces apoptosis via up- and downregulation of Bax/Bcl-2 proteins independent of functional p53 activity Induces DNA fragmentation
	Human hepatoma (HTC) [80, 81]	Cytotoxic
	Murine hepatoma cells (Hepa1c1c7) [82–84]	Marked upregulation of multiple HSPs, such as HSP40 and HSP70HSPs Increases proteasome activity with upregulation of *β*5, a major proteasome functional protein Upregulates expressions of several proautophagic markers, including p62 and microtubule-associated protein 1 light-chain 3 (LC3)-II Suppresses cellular protein modifications by 4-hydroxy-2-nonenal (HNE) Confers resistance to toxicity of HNE via p62 induction Induces ubiquitination and aggregation of cellular proteins Activates ubiquitin-proteasome system and autophagy
	Normal human liver cells (Chang) [79]	Inhibits cell growth with an IC_50_ value of 10.96 ± 0.059 *μ*g/mL
	Normal rat liver epithelial cells (RL34) [85]	Activates phase II drug metabolizing enzymes, such as GST (glutathione S-transferase), epoxide hydrolase, and hemeoxygenase via the transcription factor Nrf2 dependent pathway
	Normal human liver cells (WRL-68) [86]	Not cytotoxic
Cervical	Human cervical cancer (HeLa) [87–89]	Causes growth inhibition and induces apoptosis Decreased level of IL-6 secretion and membrane bound IL-6 receptor Induces G2/M cell cycle arrest
Colon	Human colonic adenocarcinoma (Caco-2, Colo320DM, and HT-29) [61]	Markedly induces expressions of interleukin (IL)-1*α*, IL-1*β*, IL-6, and tumor necrosis factor (TNF)-*α*
	Human colonic adenocarcinoma (LS174T, LS180, COLO205, COLO320DM) [61]	Inhibits cell proliferation in dose-dependent manner
	Normal human colon fibroblast (CCD-18Co) [61]	Not cytotoxic at a concentration of 13 *μ*M
Colorectal	Human colorectal carcinoma (HCT116) [90, 91]	Enhances TRAIL-induced apoptosis Causes activations of caspase-8, caspase-9, caspase-3 and PARP in combination with TRAIL Induces expression of TRAIL receptors DR4 and DR5 Downregulates expression of antiapoptotic protein c-FLIP Causes activation of ERK in time-dependent manner
	Human colon carcinoma (HCT-116) [76]	Induces apoptosis
Bile duct	Poorly differentiated adenocarcinoma (KKU-100), squamous cell carcinoma (KKU-M139), moderately differentiated adenocarcinoma (KKU-M156), adenosquamous carcinoma (KKUM213), and moderately differentiated adenocarcinoma (KKU-M214) [92]	ZER derivatives (5, 10, 14, and 20) showed antiproliferative activity
Breast	Human breast adenocarcinoma cell lines (MCF-7 and MDA-MB 231) [68, 90] Human breast benign cell line (MCF-10A) [76]	G2/M phase cell cycle arrest Downregulates cyclin B1, cyclin-dependent kinase 1, Cdc25C, and Cdc25B and Bax/Bak-mediated apoptosis Induces significant expression of DR4 Activation of Bax and Bak
		Not cytotoxic
Ovarian	Human ovarian cancer (Caov-3) [59]	Causes growth inhibition and induces apoptosis Decreases level of IL-6 secretion and membrane bound IL-6 receptor Induces G2/M cell cycle arrest
	Normal Chinese hamster ovarian cells (AS52) [61]	Suppresses tumor promoter 12-O-tetradecanoylphorbol-13-acetate- (TPA-) induced superoxide anion (O_2_^−^) generation from xanthine oxidase (XO)
	Normal Chinese hamster ovary cells (CHO) [93]	High concentrations produce genotoxic and cytotoxic effects (40–80 µM)
Pancreatic	Human pancreatic carcinoma (PaCa) [94]	Novel inhibitor of Jak2/Stat3, which inhibits promigratory gene expression, growth, and migration of pancreatic cancer cells
	Human pancreatic cancer (PANC-28, MIA PaCa-2, and AsPC-1) [64]	Inhibits CXCL12-induced invasion of pancreatic tumor cells
	Human pancreatic carcinoma (PANC-1 and SW1990) [95]	Time-dependent inhibition of cell viability induces apoptosis
	Human pancreatic carcinoma (PaCa) [96]	Inhibits PaCa-associated angiogenesis through the inhibition of NF-*κ*B and NF-*κ*B-dependent proangiogenic gene products
Lung	Human nonsmall cell lung carcinoma (H1299 cells) [63, 90]	Enhances TNF-induced cytotoxicity and potentiates apoptosis Inhibits TNF-induced I*κ*B*α* protein degradation and phosphorylation Inhibits TNF-induced phosphorylation of p65 protein Suppresses TNF-induced invasion activity
	Human small cell lung carcinoma (NCI-H187) [97]	Inhibits monomeric form of the HSP 27 protein ZER derivative (parent alcohol 8) induces strong cytotoxicity
Kidney	Human embryonic kidney carcinoma cell line (A293 cells) [63]	Inhibits cell growth
	Bovine normal kidney cell line (MDBK) [79]	Inhibits cell growth with an IC_50_ value of 10.02 ± 0.03 *μ*g/mL
	Human kidney embryonic cells (HEK 293) [98]	ZER derivative (parent alcohol 8) could protect irradiation induced cell apoptosis and DNA damage, at least partly, via activation of Keap1/Nrf2/ARE pathway
	Normal African green monkey kidney cells (Vero) [97]	Nonsignificant cytotoxicity with IC50 of 30 µM.
Brain	Human brain malignant glioma (GBM8401) [99]	Induces human glioblastoma multiforme cell apoptosis via inhibition of the IKK*α*-Akt FOXO1 cascade and activation of caspase-3
	Human brain malignant glioma (U87MG) [99]	Significantly decreases cell viability at the concentration of 30 and 50 *μ*M
Prostate	Human adenocarcinoma (DU145) [90]	Induces cytotoxicity and significant PARP cleavage Effectively blocks Jak2/STAT3-mediated signaling pathways Induces nonsignificant expression of DR4
	Human adenocarcinoma (PC3) [90]	Induces nonsignificant expression of DR4
Stomach	Human gastric adenocarcinoma (AGS) [100]	Inhibits tumor angiogenesis via reduction of VEGF production and NF-*κ*B activity
Oral	Human oral cancer (KB) [97]	ZER derivative (parent alcohol 8) induces strong cytotoxicity
Headand neck	Human squamous cell carcinomas (SCC4) [64]	Suppresses CXCR4 expression and cancer invasion and metastasis
	Human squamous cell carcinoma (LICR-LONHN5) [63]	Inhibits activation of NF-*κ*B and NF-*κ*B regulated gene expression Suppresses I*κ*B*α* kinase activity, phosphorylation, and degradation Suppresses p65 phosphorylation, nuclear translocation, and acylation
Pharynx	Human squamous cell carcinoma (FaDu) [63]	Inhibits NF-*κ*B and I*κ*B*α* kinase activation Suppresses antiapoptotic and metastatic gene expression Upregulates apoptosis and downregulates cancer invasion
Bone	Mouse macrophage (RAW 264.7) [68]	Inhibits RANKL-induced NF-*κ*B activation through inhibition of activation of IKBA kinase, IKBA phosphorylation, and IKBA degradation Suppresses RANKL-induced differentiation of an osteoclast precursor cells to osteoclasts Inhibits osteoclastogenesis induced by RANKL and tumor (RAW264.7) cells after incubation in the presence of MDA-MB-231 cells or U266 cells for 24 h, then exposed to ZER for 5 days, and finally stained for TRAP expression) Potential therapeutic agent for osteoporosis and cancer-associated bone loss

**Table 3:** In Vivo biological effects of zerumbone.

Organ	Animal model	ZER route	Biological effect of ZER
Cervix	Female BALB/c mice [88, 89]	Intraperitoneal injection	Suppresses cervical intraepithelial neoplasia in female Balb/c mice prenatally exposed to diethylstilbestrol (DES) Reduces the expression of cell proliferation marker PCNA in dose dependent manner Causes overexpression of proapoptotic protein Bax Suppresses Bcl-2 specific mRNA expression Inhibits progression of cervical dysplasia from becoming more severe dysplasia (CIN 3) and suppresses level of serum IL-6
Colon	Male Sprague Dawley rats [101]	Oral dose	Suppresses azoxymethane- (AOM-) induced colon cancer using aberrant crypt foci (ACFs) as a preneoplastic marker
	Male ICR mice [102]	Oral dose	Inhibits multiplicity of colonic adenocarcinomas induced by azoxymethane (AOM) Suppresses colonic inflammation in dose-dependent manner Inhibits cancer proliferation, potentiates apoptosis, and suppresses NF-*κ*B and HO-1 expressions
	Female ICR mice [103]	Oral dose	Suppresses acute ulcerative colitis (UC) induced by dextran sodium sulfate (DSS) Significantly lowers levels of inflammatory biomarkers IL-1*β*, TNF-*α*, and PGE_2 _in colonic mucosa Suppresses expression of inflammatory cytokines, TNF, and IL-1*β* in LPS/IFN-*γ*
	Male F344 rats [104]	Oral dose	Reduces development AOM-induced colonic aberrant crypt foci Reduces expression of COX-2 and prostaglandins in colonic mucosa Reduces number of AgNORs in colonic crypt cell nuclei
Liver	Male Sprague Dawley rats [105]	Intraperitoneal injection	Protects rat liver from carcinogenic effects of DEN and AAF Lowers serum ALT, AST, AP, and AFP concentrations Lowers concentration of GSH in hepatic tissue Lowers expression of PCNA in the rat liver Increases Bax and decreases Bcl-2 protein expression in the liver
	Male Sprague Dawley rats [106, 107]	Oral dose	Suppresses fatty liver formation induced by overdosage of ethanol Prevents necrosis of liver tissues after administration of overdosage of paracetamol Reduces levels of liver ALT, AST, and ALP at 24 h after administration of overdosage of paracetamol
	Male golden Syrian hamsters [108]	Oral dose	Attenuates nonalcoholic fatty liver disease Improves insulin sensitivity, decreases lipogenesis, and increases lipid oxidation
	Male Sprague Dawley rats [82]	Oral dose	Upregulates heat shock protein expressions in the liver Confers thermoresistant phenotype
Lung	Female A/J mice [102]	Oral dose	Significantly inhibits multiplicity of lung adenomas induced by 4-(Nmethyl-N-nitrosamino)-1-(3-pyridyl)-1-butanone (NNK) Inhibits cancer proliferation, potentiates apoptosis, and suppresses NF-*κ*B and HO-1 expressions
Breast	Female Sprague Dawley rats [109]	Intraperitoneal injection	Inhibits tumor growth via Wnt pathway in LA-7 bearing rats
	Female severe combined immune deficient (SCID) mice [76]	Intraperitoneal injection	Retards growth of orthotopic MDA-MB-231 xenografts in association with apoptosis induction and suppression of cell proliferation (Ki-67 expression)
	Female BALB/c nu/nu mice [68]	Intraperitoneal injection	Decreases osteolytic bone metastasis in MDA-MB-231 bearing athymic nude mice dose dependently
Blood	WEHI-3B bearing male BALB/c mice [70]	Oral dose	Induces apoptosis via the mitochondrial intrinsic pathway Increases expression of Bax, Cyt-c, and PARP and decreases the expression of Bcl-2
	CDF mice [69]	Intraperitoneal injection	Significantly prolongs life of P-388D1-bearing CDF mice
Skin	C57 BL/6 male mice [77]	Intraperitoneal injection	Significantly reduces tumor mass and lung metastasis in B16-F0 bearing mice through the activation of Akt and MAPK and inhibition of NF-*κ*B activity
	ICR mice [110]	Topical application	Suppresses 7,12-dimethylbenz[*α*]anthracene (DMBA) and TPA-induces initiation and promotion of skin tumor formation Enhances expression of antioxidative and phase II xenobiotics metabolizing enzymes manganese superoxide dismutase (MnSOD), glutathione peroxidise-1 (GPx-1), glutathione S-transferase-P1 (GST-P1), and NAD (P) H quinine oxidoreductase (NQO1) mRNA in the epidermis Suppresses TPA-induced COX-2 expression and phosphorylation of ERK1/2 Suppresses TPA-induced leukocyte maturation and dermal infiltration as well as activation stages of skin tumors
	Female HR-1 hairless mice [78]	Topical application	Induces HO-1 expression through activation of Nrf2
Paw	Mice [24]	Intraperitoneal injection	Inhibits carrageenan-induced paw edema dose dependently Suppresses granulomatous tissue formation in cotton pellet-induced granuloma test
Eye	ICR mice [111, 112]	Oral dose	Protects mouse cornea from ultraviolet B- (UVB-) induced inflammatory photokeratitis Inhibits NF-*κ*B, iNOS, and TNF-*α* expressions Abrogates nuclear translocation of NF-*κ*B Reduces malonyldialdehyde (MDA) accumulation and increases GSH and glutathione reductase levels Protects mice cornea from UVB-induced cataractogenesis
Pancreas	Male Wistar rats [113]	Oral dose	Suppresses cholecystokinin octapeptide- (CCK-8-) induced acute pancreatitis Significantly reduces serum amylase and lipase activities Reduces cytosolic IL-6 and TNF-a and increases cytosolic I*ᴋ*B*α* concentration Reduces iNOS and Mn- and Cu/Zn-superoxide dismutase activities Significantly reduces pancreatic weight to body weight ratio
	Male SPF Wistar rats [114]	Intravenous injection	Attenuates severity of acute necrotizing pancreatitis induced by sodium taurocholate and pancreatitis-induced hepatic injury, via inhibition of NF-*κ*B activity and downregulation of ICAM-1 and IL-1*β* expressions
Bone	Male Sprague Dawley rats [115]	Oral dose	Reduces inflammatory process in collagen-induced osteoarthritis (OA) Significantly reduces number of major histocompatibility complex type II cells (MHC) expression in the affected synovial membrane Reduces the number of antigen presenting type A cells presented during arthritis
	Male Sprague Dawley rats [116, 117]	Oral dose	Produces chondroprotective effects in MIA-induced knee osteoarthritis Improved immunoreactivity of neuropeptides Improves density of protein gene products (PGP), calcitonin gene-related peptide (CGRP), and neuropeptides-Y (NPY) immunoreactive nerve fibers Reduces the level of PGE_2_Produces induction of cytochrome P450 and cytosolic GST
Miscellaneous	Male ICR mice [118]	Intraperitoneal injection	Produces pronounced antinociception against chemical models of nociception through L-arginine-nitric oxide-cGMP-PKC-K+ ATP channel pathways, the TRPV1, and kinin B2 receptors
	Male BALB/c mice [119]	Intraperitoneal injection	Produces significant peripheral and central antinociceptive effects when assessed in acetic acid-induced abdominal writhing and hot-plate test models
	Female and male BALB/c mice [120]	Oral dose	No toxic effects to liver and renal tissues Does not cause significant change in hematological and serum biochemical parameters
	Female and male ICR mice [121]	Intraperitoneal injection	Does not cause mortality or change in the general condition, growth, organ weights, hematology, serum biochemistry, or histopathology after a single dosage of 500 mg/kg or multiple dosage of f 5, 25, and 50 mg/kg for a period of 28 days
	Female Sprague Dawley rats [122]	Single intraperitoneal injection	Not toxic to liver and renal tissues at dose of 100–200 mg/kg Produces severe renal and hepatic damage at a dose of 500 mg/kg with increased serum creatinine, BUN, liver enzymes (ALT, ALP, and GGT), and MDA concentrations Does not cause mortality at 100, 200, 500, and 1000 mg/kg Causes 20 and 40% death for animals receiving 1500 and 2000 mg/kg, respectively Causes 100% death in animals receiving 2500 and 3000 mg/kg
	Male Sprague Dawley rats [71, 74]	Intraperitoneal injection	Induces significant increase in the frequency of micronuclei in polychromatic erythrocytes (PCEs) at dose 1000 mg/kg after 24-hour injection Inhibits cell proliferation and causes cytotoxicity in the rat bone marrow
	Female Sprague Dawley rats [123]	Intraperitoneal injection	Beneficial in cisplatin-induced renal dysfunction, toxicity, and organ damage via preservation of antioxidant glutathione and prevention of lipid peroxidation Attenuates cisplatin, decreases renal GSH, and increased MDA levels
	Male New Zealand white rabbits [124]	Oral dose	Significantly averts and decreases early atheroma plague formation and development via reduction in monocytes and/or macrophages migration, aggregation, and smooth muscle cells proliferation in rabbits fed on cholesterol-rich diet Repairs endothelial dysfunction resulting from hyperlipidemia in rabbit atherosclerosis model
	Male golden Syrian hamsters [125]	Oral dose	Improves dyslipidemia by modulating the genes expression involved in the lipolytic and lipogenic pathways of lipids metabolism Decreases hepatic mRNA levels of fatty acid synthase, malic enzyme, sterol-regulatory element binding protein, and 3-hydroxy-3-methyl-glutaryl-CoA reductase
	Male Wistar rats [20]	Oral dose	Ameliorates streptozotocin-induced diabetic nephropathy (DN) by reducing the hyperglycemia-induced inflammatory response Decreases infiltration of macrophages, IL-1, IL-6, and TNF-*α* produced by p38 mitogen-activated protein kinase activation
